# System on Chip (SoC) for Invisible Electrocardiography (ECG) Biometrics

**DOI:** 10.3390/s22010348

**Published:** 2022-01-04

**Authors:** Francisco de Melo, Horácio C. Neto, Hugo Plácido da Silva

**Affiliations:** 1 Instituto de Telecomunicações (IT), 1049-001 Lisbon, Portugal; 2 Instituto Superior Técnico (IST), Universidade de Lisboa (UL), 1050-049 Lisbon, Portugal; hcn@inesc-id.pt; 3 Instituto de Engenharia de Sistemas e Computadores (INESC)—Investigação e Desenvolvimento (ID), 1000-029 Lisbon, Portugal

**Keywords:** electrocardiography identification, binary neural networks, system-on-a-chip

## Abstract

Biometric identification systems are a fundamental building block of modern security. However, conventional biometric methods cannot easily cope with their intrinsic security liabilities, as they can be affected by environmental factors, can be easily “fooled” by artificial replicas, among other caveats. This has lead researchers to explore other modalities, in particular based on physiological signals. Electrocardiography (ECG) has seen a growing interest, and many ECG-enabled security identification devices have been proposed in recent years, as electrocardiography signals are, in particular, a very appealing solution for today’s demanding security systems—mainly due to the intrinsic aliveness detection advantages. These Electrocardiography (ECG)-enabled devices often need to meet small size, low throughput, and power constraints (e.g., battery-powered), thus needing to be both resource and energy-efficient. However, to date little attention has been given to the computational performance, in particular targeting the deployment with edge processing in limited resource devices. As such, this work proposes an implementation of an Artificial Intelligence (AI)-enabled ECG-based identification embedded system, composed of a RISC-V based System-on-a-Chip (SoC). A Binary Convolutional Neural Network (BCNN) was implemented in our SoC’s hardware accelerator that, when compared to a software implementation of a conventional, non-binarized, Convolutional Neural Network (CNN) version of our network, achieves a 176,270× speedup, arguably outperforming all the current state-of-the-art CNN-based ECG identification methods.

## 1. Introduction

Conventional biometric recognition methods require the person to pass the finger through a reader (e.g., fingerprint), to talk to a microphone (e.g., voice recognition), or to look at a video camera (e.g., face or iris recognition). Such methods have intrinsic security limitations, as they can be affected by light or ambient noise, and can be easily “fooled” by artificial replicas (e.g., silicone finger replicas, audio recordings or pictures). Such caveats have driven researchers in the field to explore other alternatives [1,2]. ECG signals have demonstrated particularly advantageous properties, becoming a practical alternative for real-world deployment with modern approaches that addressed the usability constraints of this modality.

In particular, invisible ECG enables signal acquisition, with the sensors integrated in everyday use objects, without the user having to go through complex procedures like in conventional ECG hospital equipment [3]. There has been an increasing number of applications where invisible ECG allows the creation of a highly granular ECG medical history, useful for prevention and monitoring of cardiovascular diseases. In many use cases, everyday use objects are shared between multiple people, reason for which an ECG identification is required to separate the users’ ECG record history. Possible applications include ECG monitoring in sanitary facilities [4] and in vehicles through sensors integrated in the steering wheel [5]. Considering that the ECG sensor is already integrated in elements of the target application, a major advantage of using ECG biometrics is the fact that it does not require any additional action from the user. These ECG-enabled devices often need to associate the collected signals with the corresponding user on the edge, with small size, low throughput, and power constrains (e.g., battery operation), thus needing to be both resource and energy-efficient. This system implements a BCNN, the ECG-ID-BNet [6], in a SoC Field-Programmable Gate Array (FPGA) targeting the deployment in an invisible ECG acquisition system for sanitary facilities [4], in an invisible ECG paradigm. As described in [6], 3 heartbeats are required to perform an accurate biometric identification based on invisible ECG signals, reason for which a latency requirement of $t_{r}=3\times \frac{60}{150}=1.2$ seconds (s) for the whole identification process was imposed, in order for the system to achieve real-time identification.

The rest of the paper is organized as follows. Section 2 provides an overview of current RISC-V processors and current CNN acceleration techniques. Section 3 introduces the SoC and describes the hardware platform. Section 4 outlines the hardware accelerator of ECG-ID-BNet. Section 5 summarizes the experimental results obtained. Finally, Section 6 concludes this work and presents its main outcomes and future work.

## 2. State-of-the-Art

### 2.1. RISC-V Processors

Several processors were considered for this work, namely microcontrollers with *ARM* and *Xtensa* (e.g., ESP32) based cores. However, due to energy constrains derived from the project’s application domain, an FPGA was targeted as the preferred hardware platform. The RISC-V Instruction Set Architecture (ISA) was first developed in 2010, at UC Berkeley, and has been gaining popularity in both academia and industry [7]. The supported ISA is fundamental to a CPU, since, together with the compiler, it links the hardware and the software by mapping the high level software constructs into low level instructions that the CPU can and will execute [8]. Compared with other architectures, the RISC-V provides a number of advantages that makes it especially attractive for development [9]:

- The open-source nature allows any interested entity access to the source Intellectual Property (IP) of the cores without any licensing issue;
- Even though the development of the ISA is open-source, its major features are already well defined and stable, which also attracts software development;
- Additional functionalities are available through a set of extensions, which are also well defined after stabilization;
- Due to its modular nature, the ISA is suitable for both high performance and low power integrated circuit applications. Additionally, specialized application processors that feature dedicated accelerators are also supported by the ISA.

This rising support is mainly due to it’s open-source characteristic and the advantages listed above, which enables developers to create their own cores following the RISC-V ISA guidelines. However, the other core attributes must be defined by the developer. Therefore, a wide range of different memory interfaces (examples: *AXI* and *Wishbone*) and configurations is used to add peripherals to the core, access memories or control execution. One processor can use separate AXI interfaces to access instruction or data memories, while another core (i.e., processor) uses a single Wishbone bus to access both [9]. This heterogeneity complicates the choice of the right processor for a specific use-case, among all the options core landscape. Due to the complexity of the implementations, many cores, although supported by variety of toolchains, will not work out-of-the-box in the specific user target platform. In the scope of this work we focused in open-source, FPGA-optimized implementations. Other requirements were introduce in order to narrow down the number of cores explored, such as:

- As some singularities of ASIC-targeted designs, for instance the memory generator usage, might make them unusable for an FPGA implementation, these kind of designs were discouraged for this work;
- Cores must be able to support the recent versions of the ISA in order to be targeted by the recent versions of the software toolchain.

Following these criteria, some notable cores have been summarized in Table 1. This table displays some open-source RISC-V processors and some of the most important features when selecting a processor for a given application.

In [9], the authors performed a thorough evaluation of the performance and efficiency of some of the cores listed in Table 1. Their selection of cores for a close evaluation consists of: *Piccolo and Flute*, *Orca*, *PicoRV32*, *SweRV*, *Taiga* and *VexRiscv*, also, as a “gold standard”, they used a 5 and a 8 stage proprietary cores. As these cores have configurations that differ from one another, the authors were careful to make the same configurations for all cores, allowing a fair comparison between them (e.g., cache was disabled for all that support this feature). To evaluate them in real hardware, the authors also used the so called TaPaSCo [11], a system-on-chip generator for FPGAs, to easily generate bitstreams for a variety of platforms without having to deal with low level integration details. The hardware used consisted in an array of Xilinx made FPGA platforms: (1) AU250; (2) PYNQ; (3) VC709; (4) ZCU102. The chosen evaluation metrics were *Single Core Performance* and, since the target hardware are FPGAs, *Resource Utilization*.

To evaluate the performance, there are many widely used benchmarking options, most notably, there is the *CoreMark* and the *DMIPS*. For the latter, the results were quite surprising. The two proprietary cores (the authors did not mentioned their names nor their authors), which in theory had a high level of optimization, actually performed quite poorly when compared with some open source cores. With this benchmark, we can also observe that some cores have less Instructions Per Clock (IPC) but achieve reasonable results with higher working frequencies, such as the PicoRV32. The results were similar in both benchmarks; the most performing cores were the Taiga, VexRiscv and the Orca.

In terms of resource usage the results were also surprising. The cores that actually had a better performance were the ones that also consumed the least FPGA resources. Meaning that those cores, especially Taiga, Orca and VexRiscv are highly optimized in terms of performance and resource usage.

### 2.2. FPGA-Based CNN Accelerator

This section presents methodologies from previous works that aim to accelerate the inference of CNNs and recent approaches to ECG identification. The first section focuses on conventional CNNs and next section (Section 2.3) specifically targets BCNNs and ECG identification.

FPGA-based CNN accelerators aim to improve inference performance by parallelizing the CNN forward propagation. There are 6 main ways to parallelize a CNN’s inference [12]: intra convolution parallelism, inter convolution parallelism, inter feature map parallelism, intra feature map parallelism, inter layer parallelism and batch parallelism. This section will review previous works based on the implementation of 2D CNNs.

Some previous FPGA-based CNN accelerators [13,14,15] follow a standard architecture that entails: an external memory, used to hold the CNN’s parameters and input/output feature maps; an input and output buffer to cache the input and output feature maps; a Processing Element (PE) that processes the operations required to compute the outputs using the cached inputs and the weights that are maintained in a PE’s internal buffer; and a controller which controls the overall execution.

In works such as [13,14], a PE performs concurrent Multiply and Accumulate (MAC) operations to solve a single 2D convolution. This is an example of intra convolution parallelism.

The authors of [13] take advantage of inter convolution parallelism. Their accelerator implements a PE that is capable of processing multiple input feature maps, by summing the result of multiple concurrent 2D convolutions with the objective of computing a single 3D convolution necessary to produce an element of a output feature map.

By having an array of PEs, where each PE is designed to compute a single output feature map at the time, the approaches found in [13,15,16] are able to concurrently process multiple output feature maps, which is a form of inter feature map parallelism.

In [17], the authors implemented all layers in a pipelined structure that enables the execution of all layers concurrently, while requiring a substantial amount of FPGA resources. This is an example of inter layer parallelism. In the same work, the authors propose a *divide and conquer* strategy in the computation of fully connected layers that, if executed all at once, require a substantial amount of memory to hold all the operands. They divide the operation into multiple simple sub-convolutions, whose results can be accumulated to get the final result; this is an example of batch parallelism.

In [18], the authors note that in modern CNN architecures, such as [19], in deeper convolutional layers, the number of input/output channels surpass the actual input/output feature map size. In such CNNs, the authors argue that intra feature map parallelism is preferred over inter feature map parallelism.

### 2.3. BCNN-Based Optimization

Quantization of the network’s parameters is a popular practice to save memory usage and increase computational performance. The parameters are typically represented by 32-bit floating-point values, however, the hardware required to process floating-point data is much more complex and slower, comparatively to what is needed to handle integer data [20]. An approach commonly found in the literature is to quantize floating point values to 8- or 16-bit integer values, but a more extreme quantization can be performed. Binary Neural Network (BNN)s, first proposed by Courbariaux et al. in [20], introduced the concept of constraining the activations and the parameters of a Neural Network (NN) to either +1 or −1 [20], allowing a 1-bit representation. This new quantization paradigm helps minimize the memory footprint.

FPGA implementations of BCNN follow a similar architecture as conventional CNNs. The main difference lies with the datapath structure of each PE, where the multipliers and adder trees seen in conventional CNN accelerators are replaced with logic that implements the *XNOR Dot Product* (XNP) operation (described in [20]). In [21], the authors propose a pipelined PE datapath consisting of four stages: *XNOR*, *popcount*, accumulation and Batch Normalization (BN) + binarization. Works such as [16,18,21,22] employ intra and inter convolution parallelism, in addition to inter feature map parallelism.

The novel ECG-ID-BNet [6], a BCNN that implements ECG classification, was used as the ECG identification method and it is detailed in Table 2, where unit refers to a sequential stack of a convolutional/fully connected layer, a max pooling layer (if present) and a batch normalization layer. The BCNN was evaluated on real-world data from the Physionet Computing in Cardiology Challenge 2017 dataset [23] (containing 8528 ECG recordings lasting from 9 to just over 60 seconds, acquired at the hand palms using the AliveCore (https://www.alivecor.com/, accessed on: 1 October 2021) device and from our own dataset collected containing ECG data collected at the thighs using an experimental device integrated in a toilet seat cover [4] (further described in [6]).

From the AliveCore dataset we randomly selected ECG recordings classified as normal and with 60 s duration for 50 different subjects, while our dataset contains ECG recordings lasting approximately 180 seconds for 10 different subjects. The best results with ECG-ID-BNet were obtained using 4 convolutional and 1 fully-connected units, and it managed to achieve a 100±0(%) and 99.3±3.2(%) accuracy on the AliveCore dataset and our dataset, respectively. Comparatively with the state-of-the art [24] these results exhibit similar accuracy, hence demonstrating that the BCNN approach does not degrade the quality of the recognition, with the advantage of improved computational performance.

## 3. Software Implementation

In an effort to deploy the ECG-ID-BNet *on the edge*, the IOb-SoC (https://github.com/IObundle/iob-soc, accessed on: 1 October 2021) was implemented in a FPGA, as it provides a convenient and efficient infrastructure to create FPGA SoC. In this section, the software implementation is described.

### 3.1. Hardware Platform

IOb-SoC is an open-source RISC-V based SoC platform written in Verilog. This platform is composed of a RISC-V soft-core, the PicoRV32 (https://github.com/cliffordwolf/picorv32, accessed on: 1 October 2021), an internal SRAM subsystem, an optional external memory interface and peripherals. It implements the RISC-V RV32IMC instruction set [7] and it can be programmed with the RISC-V GNU Compiler Toolchain (https://github.com/riscv-collab/riscv-gnu-toolchain, accessed on: 1 October 2021). The hardware platform selected to implement the IOb-SoC was the FPGA development board *Basys3* (https://digilent.com/reference/programmable-logic/basys-3/start, accessed on: 1 October 2021), which features a Xilinx Artix-7 XC7A35T FPGA whose main characteristics are shown in Table 3.

### 3.2. ECG-ID-BNet Memory Usage

As the target hardware platform to deploy the NN is a resource constrained embedded system, the memory usage of the NN must be carefully monitored. A memory usage study of ECG-ID-BNet, whose architecture is detailed in Table 2, was conducted in order to define the memory necessary to: (1) hold the network’s parameters; and (2) hold the input/output feature maps of each layer.

In Table 4, each unit’s memory usage is summarized. The convolutional units have a considerably lower number of parameters when compared to the fully connected one. As a requirement for the hardware platform, it is concluded that ECG-ID-BNet requires 57,200 Bytes of memory to hold the parameters and two buffers of at least 2784 Bytes each to hold the units’ input/output feature maps.

### 3.3. Memory Reorganization

**Figure 1:**
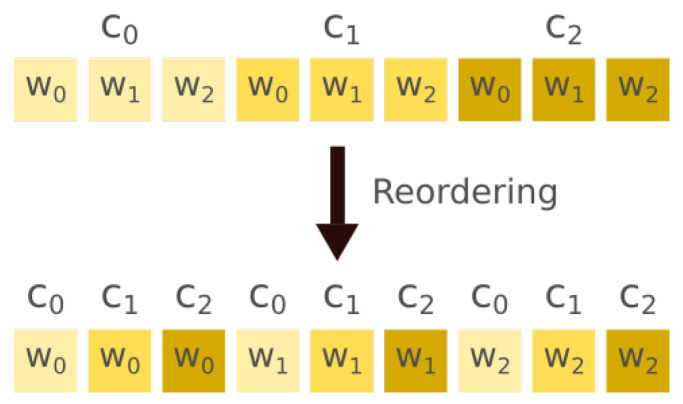
Weights reordering on a filter that expects 3 input channels and whose kernels have size 3.

The traditional storing order of a filter’s weights is (x,c), where *x* indicates the kernel width dimension and *c* the channel dimension. However, this work proposes the weight arrangement (c,x), illustrated in Figure 1.

Doing this rearrangement enables inter convolution parallelism, allowing the fast computation of a single output feature map element. Furthermore, every ECG-ID-BNet layer (see Table 2) has a 64 multiple number of input filters, with exception of the first layer. With this ECG-ID-BNet feature, it is possible to group 64 1-bit weights in 64-bit blocks. 64-bit was chosen as the size of the blocks, over other valid bit widths such as 32-bit, in order to ensure compatibility between the software application and the IP developed in Section 4. By having weights memory arrangement, it is possible to enable operand parallelism, taking advantage of the 32-bit computing of the targeted processor if the input feature maps are also in the (c,x) arrangement.

### 3.4. Execution Flow

Each unit’s output becomes the following unit’s input. This enables the adoption of two memory buffers, used in a *ping-pong* fashion, where their place alternate every unit iteration.

**Figure 2:**
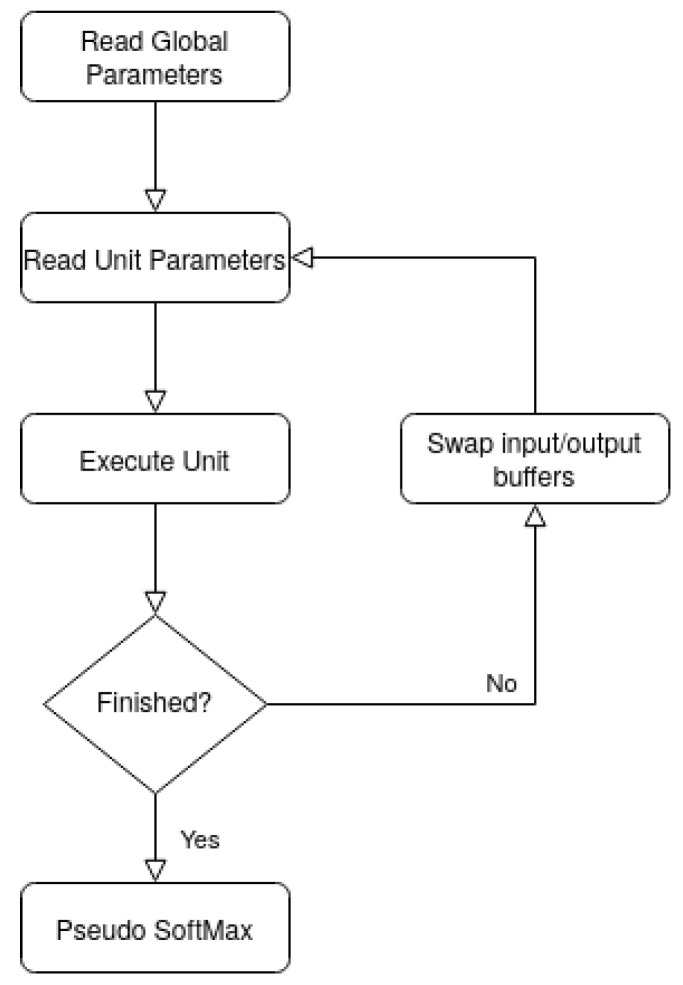
High-level workflow of the software.

Figure 2 depicts the high-level flowchart of the software application. A pre-compiled binary file with all necessary ECG-ID-BNet parameters is used for inference. The file contains: global parameters, such as number of units and convolutional kernel size; unit parameters, including the type of unit, the presence of max pooling, the unit’s relative position in the network, number of input and output feature maps, output feature map size, and the convolutional/fully connected layer’s parameters (weights and binarization thresholds for first and hidden units). A *Pseudo SoftMax*, instead of *SoftMax*, is performed on the batch normalized outputs of the last unit, where the predicted class is assigned by determining the max output value, skipping the probability distribution transformation carried out by *SoftMax*.

### 3.5. IOB-Soc Configuration

The memory requirements of ECG-ID-BNet are presented in Section 3.2. Considering the resources of the hardware platform (briefed in Table 3), it was concluded that the memory resources of the FPGA are sufficient to hold the firmware and to satisfy the BCNN’s memory needs. Hence, the external memory is not necessary. Special care was taken to the dimensioning of the SRAM since, beyond the parameters and the firmware, the program’s stack, where the input and output feature map buffers reside, also takes a considerable amount of memory and thus needs to be taken into account.

IOb-SoC’s SRAM is composed of cascaded 36 kb Block RAM (BRAM)s and the BRAMs are configured to be byte-addressable, by having the configuration 4k×9 (9-bit 4 k words) and ignoring one bit per word. Considering the sections memory sizes in Table 5, a preliminary test was conducted using a 128 kB SRAM implementation; the program ran successfully, thus validating the SRAM’s size. The final SRAM memory address mapping is described in Table 6. Since each BRAM has 4k 9-bit sized words, the total SRAM’s expected usage of BRAMs is given by $\frac{128k}{4k}=32$.

### 3.6. IOb-SoC Optimization

Contrary to the BCNN software implementation, which is able to have a speedup of 32 in all units (except the first) over a CNN implementation (see Section 3.3) due its inter convolution parallelism, a conventional CNN cannot achieve any kind of parallelism during inference in a software implementation (using only one core). Thus, a PicoRV32 software implementation of the inference of a conventional CNN version of ECG-ID-BNet would take around $t_{sw_{CNN}}=0.24+32\left(0.37+0.4+0.34+0.01\right)=36.1$ s. This implies a speedup of $\frac{t_{sw_{CNN}}}{t_{sw_{BCNN}}}=\frac{36.1}{1.36}=26.5$ of a BCNN version over a conventional CNN one.

The performance results show that a software only approach is not able to achieve real-time ECG identification, since the overall identification process (preprocessing, feature extraction and classification) needs to be under 1.2 s (see Section 1) and only the ECG-ID-BNet takes 1.36 s > 1.2 s. As such, a more efficient solution for the ECG-ID-BNet inference is needed.

## 4. IP-Core

**Figure 3:**
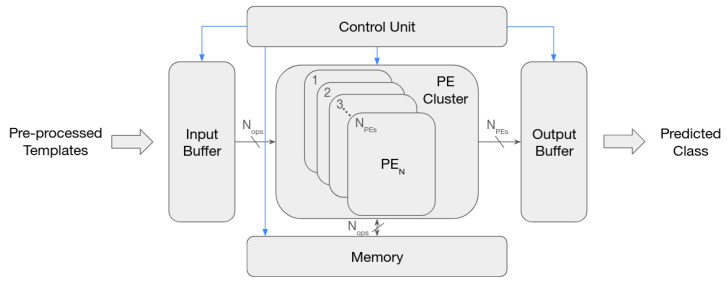
ECG-ID-BNet accelerator high-level architecture. The blue arrows represent the control signals, while the black arrows depict the datapath. $N_{ops}$ denoted the number of operations each PE processes at a time and $N_{PEs}$ denote the number of processing elements.

An IP core was designed to execute ECG-ID-BNet in the same FPGA used in Section 3. The hardware architecture (illustrated in Figure 3), selected based on the state-of-the-art review (Section 2.2), is similar to the ones developed in the works [13,14,15]. Two methods of CNN parallelism were used: (1) inter convolution, by having a PE processing $N_{ops}$ inputs, each belonging to a different feature map; and (2) inter feature map, by having a group of $N_{PEs}$ PEs concurrently computing multiple output feature maps (i.e., each PE executes one filter). These allow all PEs to process the same input feature map elements, thus reducing the required input memory bandwidth. The possible number of concurrently processed input feature maps is limited due to BRAM bandwidth, which is explored in Section 4.2.

### 4.1. Execution Flow and Control

The control unit of the proposed IP is subdivided into two units that work in parallel: (1) the parameters control unit, responsible to download the parameters of each filter from the parameters RAM into each corresponding PE internal buffer; and (2) the execution control unit that oversees the inference process by controlling the PEs and input/output feature map RAMs via control signals. Both control units are accomplished with finite state machines.

**Figure 4:**
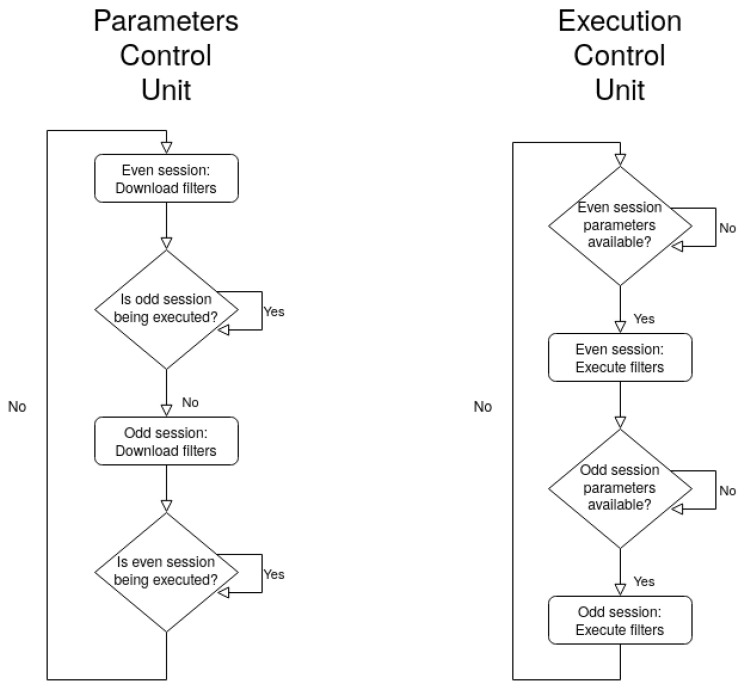
ECG-ID-BNet IP core execution flow.

In Figure 4, a depiction of the execution flow is presented. In the scope of this work “session” is defined as the computation of $N_{PEs}$ output feature maps concurrently by the PEs, thus multiple sessions are needed in order to fully execute a layer, e.g., with $N_{PEs}=32$ and $N_{out\_c}=64$, $N_{sessions}=\frac{N_{out\_c}}{N_{PEs}}=2$ are needed. Moreover, an “even” and “odd” session refer to an even numbered session (e.g., session 0) and to an odd nuber session (e.g., session 1). The ECG-ID-BNet inference starts with a setup phase, which involves downloading the first batch of $N_{PEs}$ filters, to enable the execution of the first session. After that, the execution parameters control units work concurrently in a *ping-pong* fashion: when one is working in an “even” session, the other is working in an “odd” one.

This promotes execution performance, but puts a memory constrain in the internal PE memories, since they need to be large enough to hold two sets of filters’ weights at a time. The control unit employs counters to iterate through the network’s inference iterators. The input/output memory module contains an address calculator, which uses a counter to keep track of the current output address and current output channel to compute the PEs’ outputs memory destinations.

### 4.2. Memory Specification

The target FPGA contains 50 36kb BRAMs (detailed in Table 3), and each BRAM can be configured as single-port RAM, simple dual-port RAM, true dual-port RAM, simple-port RAM or dual-port RAM [25]. The simple dual-port RAM is the configuration that enables maximum port width; due to the FPGA constraints, the BRAM’s depth×width ratio must be one of the following: 32k×1; 16k×2; 8k×4; 4k×9; 2k×18; 1k×36; or 512×72 [26].

This IP uses the internal FPGA BRAMs to hold three main data components: (1) NN parameters; (2) weights for each filter; and (3) input and output feature maps. In Table 7, a detailed ECG-ID-BNet memory usage is shown, where the memory necessary to hold the weights of each filter/neuron are indicated.

The memory holding the parameters is configured as a multiple cascaded single-port RAM. The amount of BRAMs needed to support the parameters is defined by $\frac{57,200\times 8}{36k}=12.41$ (ECG-ID-BNet’s parameters size divided by BRAM size), which gives a total of 13 BRAMs. As shown in Table 4, the two buffers must have a minimum size of 2784 bytes; this can be accomplished with a single BRAM for each of them. Similarly, in Table 7, it is possible to check that the maximum filter or neuron size is 632 bytes, and since each PE’s weights buffer must be able to hold two filters/neurons, it’s minimum size must be 632×2=1264 bytes, which can also be accomplished with a single BRAM.

Due to the fact that the weight and input/output RAMs are composed of a single BRAM, the maximum possible width, due to the FPGAs constraints, is 72 bits, unless more BRAMs are cascaded. However, that would deplete rapidly the number of BRAMs available and consequently the possible number of PEs. Using the same memory reorganization described in Section 3.3, each PE processes multi-bit blocks of inputs and weights, enabling inter convolution parallelism. Additionally, by analysing the topology of ECG-ID-BNet (Table 2), it is possible to conclude that the number of input feature maps is always a multiple of 64, with the exception of the first unit. This enables a fast and simple computing of output feature map elements by each PE, if the number of concurrently processed inputs and weights $N_{ops}$ is 64, which is possible to achieve by configuring the weights and input feature map BRAMs as 512×72 and using 64 out of the 72 bits of the configured BRAM width.

The memory generator [25] was used to generate the parameters, input feature maps, output feature maps and each of the weights RAMs. Table 8 summarizes the configurations of each RAM generated. The maximum possible number of PEs is thus limited by the amount of available BRAMs. Since the target FPGA features 50 BRAMs (detailed in Table 3), the maximum number of PEs is given by $N_{PEs_{max}}=50-15=35$. However, by inspecting the number of executing sessions it takes to compute each layer, it is possible to reduce the number of PEs to 32, without penalizing performance, as shown in Table 9.

## 5. Results

This section presents the results of the various results obtain throughout the course of this work, namely the performance results of both the software and IP core implementations. Additionally, a comparative analysis between the two implementations is made.

### 5.1. Software Implementation

With the objective of implementing the IOb-SoC in the *Basys3* development board, a synthesis and implementation were performed, where the SoC’s internal SRAM was pre-loaded with the firmware and the ECG-ID-BNet’s parameters. The IOb-SoC hardware design was completed and FPGA implementation was conducted.

The FPGA resource usage are presented in Table 10 and the clock frequency for the PicoRV32 was finalized at 100 MHz. This clock frequency was chosen given that it is near the maximum possible operating frequency, also being the same frequency of the IP-Core (detailed in Section 4), hence being beneficial for the performance profiling shown in Section 5.2. Among the BRAMs used by IOb-SoC, 1 is used for its internal bootloader and the other 32 are used for the SRAM.

The ECG-ID-BNet was executed in the IOb-SoC and every unit’s execution times were monitored; the results are presented in Table 11. A compiler optimization was tested (*O3*) and it resulted in a 40% execution speedup, when compared to a non-optimized implementation. The highest execution time is observed in the convolutional units, where the program spends 99% of the time.

### 5.2. ECG-ID-BNet IP-Core

With the purpose of evaluating the developed IP core, a synthesis was performed. A resource utilization report was made to the four main modules of the IP. The report is represented in Table 12, where the PE cluster is the module containing all $N_{PEs}=32$ PEs. It shows that, relatively to the FPGA resources, there is 19% LUT, 10% FF, 94% BRAM and 0% DSP usage. The PE cluster uses on average 103 LUTs and 109 FFs per PE. The input/output memory module, beyond the 2 BRAMs, uses logic resources for the output address calculator. No DSP slice is used because the proposed IP does not require complex computations, such as multiplications or divisions. The control unit uses 543 LUTs for its finite states machines and various counters, of which 258 are dedicated to the parameters control unit and 285 for the execution control unit. Along the synthesis, it was imposed a clock frequency constraint of 100 M Hz as a first proposition and a timing report revealed a Worse Negative Slack (WNS) of 1.1 ns.

A simulation of the IP core execution was made for performance profiling purposes. Table 13 shows the results, where the execution times are presented, per unit, alongside the software only implementation (O3 level optimized) already shown in Section 5.1. Additionally, the speedup that the IP core manages to achieve over the software only execution is also represented. In comparison with other units, the first one achieves half the speed up. This is due to the fact that each PE is only able to process a single input and weight at a time. Another notable observation is the increased inter convolution parallelism that the IP core has over the software implementation, since each PE processes 64 inputs and weights at the time, while the other processes 32 inputs and weights at a time. This fact is especially visible on the speed up difference between the first and the other convolutional units, where the first unit’s speedup is half of that of the others.

**Figure 5:**
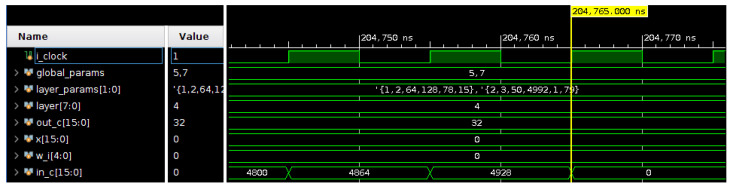
Simulation snapshot of the IP core @ 100 MHz showing the end of the computation of the last unit.

The last unit is not able to accomplish nearly as much speedup as the rest of the units. This is due to the fact that, contrary to the convolution layers, the fully connected layer’s parameters are numerous (see Table 4) and downloading them onto each PE’s internal memory takes about 5× longer than the other layers. The execution of this unit is faster than its parameters downloading process, thus, most of the time spent in this unit the execution is idle. As stated in Section 3.6, a conventional CNN version of ECG-ID-BNet would take 36.1 s to execute in a software-only paradigm. Thus, we argue that this BCNN accelerator achieves at least a $\frac{t_{sw_{CNN}}}{t_{IP_{BCNN}}}=\frac{36.1\;s}{204.8\;\mu s}=$ 176,270 speedup over the CNN PicoRV32 software implementation. In addition, a simulation snapshot of the IP’s execution is presented in Figure 5. This snapshot shows the last stages of the forward propagation of the last unit alongside the execution’s main control signals, mainly the BNN’s global parameters, the units parameters (*layer_parameters*), and the iterators’ counter values.

## 6. Conclusions

A SoC that performs ECG identification was proposed. The main components of such system were developed, namely the The RISC-V based SoC architecture, and the NN accelerator. The IOb-SoC, which integrates the PicoRV32, was configured and implemented successfully in the *Basys3* FPGA development board. The PicoRV32 was programmed with the software implementation of ECG-ID-BNet, and it was successfully executed with an execution time of 1.32 s. This software implementation already managed to show a $\frac{t_{sw_{CNN}}}{t_{sw_{BCNN}}}=\frac{36.1\;s}{1.32\;s}=27.3$ speedup over a similar conventional CNN version of ECG-ID-BNet implementation, due to the binary nature of ECG-ID-BNet.

However, software only solution proved to be insufficient to cope with the latency requirement of 1.2 s, defined in Section 1, to enable real-time ECG identification. As such, the ECG-ID-BNet IP core was designed and implemented successfully in the *Basys3* FPGA development board. It showed its ability to achieve real-time ECG identification, by running the inference of the network in only 204.8 μs, which translates to a 6641× speedup over the software implementation and a 176,270× speedup over a similar software implementation but with a CNN, non-binarized, version of ECG-ID-BNet arguably outperforming the current state-of-the-art CNN-based ECG identification methods in terms of execution time.

In summary, this work further extends the state-of-the-art with: (a) a SoC architecture with small hardware footprint capable of performing fast and low-power ECG biometrics in real time; (b) a software implementation of the inference stage of a BCNN in a size-optimized RISC-V core; and (c) an optimized IP core that performs ECG classification with a latency of 205 μs, arguably outperforming the current state-of-the-art CNN-based ECG identification methods in terms of execution time.

Although all the objectives set forth for this work were met, a number of future work opportunities have also been created, namely concerning the integration of the different peripherals of the SoC via a memory interface, such as *AXI*. As described in Section 2.1, the PicoRV32 is not the most performing core and as such, can be replaced with a more efficient one. Furthermore, it would also be interesting to evaluate the ability for the proposed SoC to generalize to other biometric modalities (e.g., voice, gait, etc.).

## Figures and Tables

**Table 1:** Properties of selected 32-bit RISC-V cores. Adapted from [10] and from own research.

Name	Pipe Line Stages	Bus Arch	HDL	SIMD	Debug Support	License	Last Update	Cache
Freedom	5	TL/AXI	Chisel	N	Y	BSD	No more Support	Y
ORCA	5	WB AXI	VHDL	N	N	BSD	2019	Y
RI5CY	4	AXI	Verilog	Y	Y	Solder pad	2021	N
Zero-Riscy	2	AXI	Verilog	N	Y	Solder pad	2018	N
OPenV	3	AXI	Verilog	N	N	MIT	2018	N
VexRiscv	5	AXI	Spinal HDL	Y	Y	MIT	2021	Y
Roa Logic RV12	6	AHB WB	Verilog	N	Y	Non	2018	Y
SCR1	4	AXI	Verilog	N	Y	Solder pad	2021	N
Humming Birdv2 E200	2	AXI	Verilog	N	Y	Apache	2021	Y
Shakti	3	AXI	Bluespec	N	N	BSD	2019	Y
ReonV	7	AHB	VHDL	N	Y	GPL v3	2018	Y
Pico RV32	0	AXI	Verilog	N	N	ISC	2020	N
SweRV EH1	9	AXI	Verilog	N	Y	Apache	2021	Y
Taiga	3	AXI	Verilog	N	N	Apache	2020	Y
Potato	5	WB	VHDL	N	N	BSD	2018	Y
Flutte	5	AXI	Verilog	N	Y	Apache	2021	Y
Piccolo	3	AXI	Verilog	N	Y	Apache	2021	Y

**Table 2:** ECG-ID-BNet architecture. Extracted from [6].

	# Filters/Neurons	Input Fmaps (c,x)	Output Fmaps (c,x)	Max Pooling
**Convolutional Unit 1**	128	(1,180)	(128,174)	N
**Convolutional Unit 2**	64	(128,174)	(64,168)	N
**Convolutional Unit 3**	128	(64,168)	(128,162)	N
**Convolutional Unit 4**	64	(128,162)	(64,78)	Y
**Fully connected Unit**	50	(4992,1)	(50,1)	N

**Table 3:** XC7A35T FPGA main specifications.

LUTs	FFs	36 kb BRAMs	DSP Slices
33,280	41,600	50	90

**Table 4:** ECG-ID-BNet memory usage summary.

	Input Fmaps (Bytes)	Output Fmaps (Bytes)	Parameters (Bytes)
**Convolutional Unit 1**	180	2784	2048 (3.6%)
**Convolutional Unit 2**	2784	1344	7680 (13.4%)
**Convolutional Unit 3**	1344	2592	8192 (14.3%)
**Convolutional Unit 4**	2592	624	7680 (13.4%)
**Fully Connected Unit**	624	200	31,600 (55.2%)
	**Max**: 2784		**Total**: 57,200 (100%)

**Table 5:** IOb-SoC ECG-ID-BNet SRAM section memory size.

Section	Memory Size (Bytes)
Firmware	28,024
Stack	Unknown
Model Parameters	57,200

**Table 6:** IOb-SoC ECG-ID-BNet SRAM address mapping.

Section	Address Range
Firmware	0x00000–0x06D78
Stack	0x06D79–0x0FFFF
Model Parameters	0x10000–0x1FFFF

**Table 7:** ECG-ID-BNet memory usage per filter/neuron in each unit summary.

	# Filters/Neurons	Parameters (Bytes)	Filter/Neuron Memory Size (Bytes)
**Convolutional Unit 1**	128	2048 (3.6%)	16
**Convolutional Unit 2**	64	7680 (13.4%)	120
**Convolutional Unit 3**	128	8192 (14.3%)	64
**Convolutional Unit 4**	64	7680 (13.4%)	120
**Fully connected Unit**	50	31,600 (55.2%)	632
		**Total**: 57,200 (100%)	**Max**: 632

**Table 8:** RAM configurations summary.

	# BRAMs	RAM Configuration (depth×width)
**Parameters**	13	7168×64
**Input Feature Map**	1	512×64
**Output Feature Map**	1	512×64
**Weights**	1/PE	512×64
	**Total**: 15 + $N_{PEs}$	

**Table 9:** Number of executing sessions required to compute each ECG-ID-BNet layer, in function of $N_{PEs}$.

	# Filters/Neurons	# Sessions Required
**Convolutional Unit 1**	128	$4_{N_{PEs}=32},\;4_{N_{PEs}=35}$
**Convolutional Unit 2**	64	$2_{N_{PEs}=32},\;2_{N_{PEs}=35}$
**Convolutional Unit 3**	128	$4_{N_{PEs}=32},\;4_{N_{PEs}=35}$
**Convolutional Unit 4**	64	$2_{N_{PEs}=32},\;2_{N_{PEs}=35}$
**Fully Connected Unit**	50	$2_{N_{PEs}=32},\;2_{N_{PEs}=35}$
		**Total**: $14_{N_{PEs}=32},\;14_{N_{PEs}=35}$

**Table 10:** IOb-SoC with ECG-ID-BNet parameters and firmware pre-loaded FPGA resource utilization (relative to FPGA’s resources).

LUTs	FFs	36 kb BRAMs	DSP Slices
2233(6.7%)	1212(2.9%)	33(66%)	4(4.4%)

**Table 11:** Execution time of the ECG-ID-BNet IOb-SoC @ 100 MHz, software only implementation.

Unit	No Optimization Execution Time (s)	O3 Optimization Execution Time (s)
Convolutional Unit 1	0.3(15.6%)	0.24(17.8%)
Convolutional Unit 2	0.55(28.1%)	0.37(27.1%)
Convolutional Unit 3	0.57(29.5%)	0.4(29.2%)
Convolutional Unit 4	0.5(26%)	0.34(25%)
Fully Connected Unit	0.014(0.75%)	0.01(0.73%)
	**Total**: 1.9(100%)	**Total**: 1.36(100%)

**Table 12:** ECG-ID-BNet IP core FPGA resource utilization.

	LUTs	FFs	36 kb BRAMs
**Control Unit**	543(13.5%)	623(14.7%)	0(0%)
**Parameters Memory**	0(0%)	0(0%)	13(27.7%)
**Input/Output Memory**	158(3.9%)	102(2.4%)	2(4.3%)
**PE Cluster**	3312(82.5%)	3517(82.9%)	32(68.1%)
**Total**	4013(100%)	4242(100%)	47(100%)

**Table 13:** Performance profiling of the ECG-ID-BNet inference in the proposed IP core @ 100 MHz compared with PicoRV32 @ 100 MHz (software only implementation, optimized with O3 level).

Unit	PicoRV32 @ 100 MHz(s)	IP Core @ 100 MHz(μs)	Speedup
**Convolutional Unit 1**	0.24(17.8%)	49.6(24.2%)	4839×
**Convolutional Unit 2**	0.37(27.1%)	47.2(23%)	7839×
**Convolutional Unit 3**	0.4(29.2%)	45.6(22.3%)	8772×
**Convolutional Unit 4**	0.34(25%)	43.8(21.4%)	7763×
**Fully Connected Unit**	0.01(0.73%)	18.6(9.1%)	537×
	**Total**: 1.36(100%)	**Total**: 204.8(100%)	6641×

## Data Availability

Not applicable.
